# BACE1 inhibitor drugs in clinical trials for Alzheimer’s disease

**DOI:** 10.1186/s13195-014-0089-7

**Published:** 2014-12-24

**Authors:** Robert Vassar

**Affiliations:** Department of Cell and Molecular Biology, Northwestern University, The Feinberg School of Medicine, 303 E. Chicago Avenue, Chicago, IL 60611 USA

## Abstract

β-site amyloid precursor protein cleaving enzyme 1 (BACE1) is the β-secretase enzyme required for the production of the neurotoxic β-amyloid (Aβ) peptide that is widely considered to have a crucial early role in the etiology of Alzheimer’s disease (AD). As a result, BACE1 has emerged as a prime drug target for reducing the levels of Aβ in the AD brain, and the development of BACE1 inhibitors as therapeutic agents is being vigorously pursued. It has proven difficult for the pharmaceutical industry to design BACE1 inhibitor drugs that pass the blood–brain barrier, however this challenge has recently been met and BACE1 inhibitors are now in human clinical trials to test for safety and efficacy in AD patients and individuals with pre-symptomatic AD. Initial results suggest that some of these BACE1 inhibitor drugs are well tolerated, although others have dropped out because of toxicity and it is still too early to know whether any will be effective for the prevention or treatment of AD. Additionally, based on newly identified BACE1 substrates and phenotypes of mice that lack BACE1, concerns have emerged about potential mechanism-based side effects of BACE1 inhibitor drugs with chronic administration. It is hoped that a therapeutic window can be achieved that balances safety and efficacy. This review summarizes the current state of progress in the development of BACE1 inhibitor drugs and the evaluation of their therapeutic potential for AD.

## Introduction

### The role of β-amyloid in Alzheimer’s disease

The extracellular accumulation of amyloid plaques composed of the β-amyloid (Aβ) peptide represents one of the two defining lesions in Alzheimer’s disease (AD) brain, the other being intracellular aggregation of hyperphosphorylated tau into neurofibrillary tangles. Recent results indicate that amyloid deposition begins ~10-20 years before the onset of dementia, suggesting that cerebral accumulation of Aβ has as a critical early role in AD pathogenesis [1-3]. If so, then inhibition of Aβ accumulation in the brain may benefit AD, if given early enough during the course of the disease.

**Figure 1:**
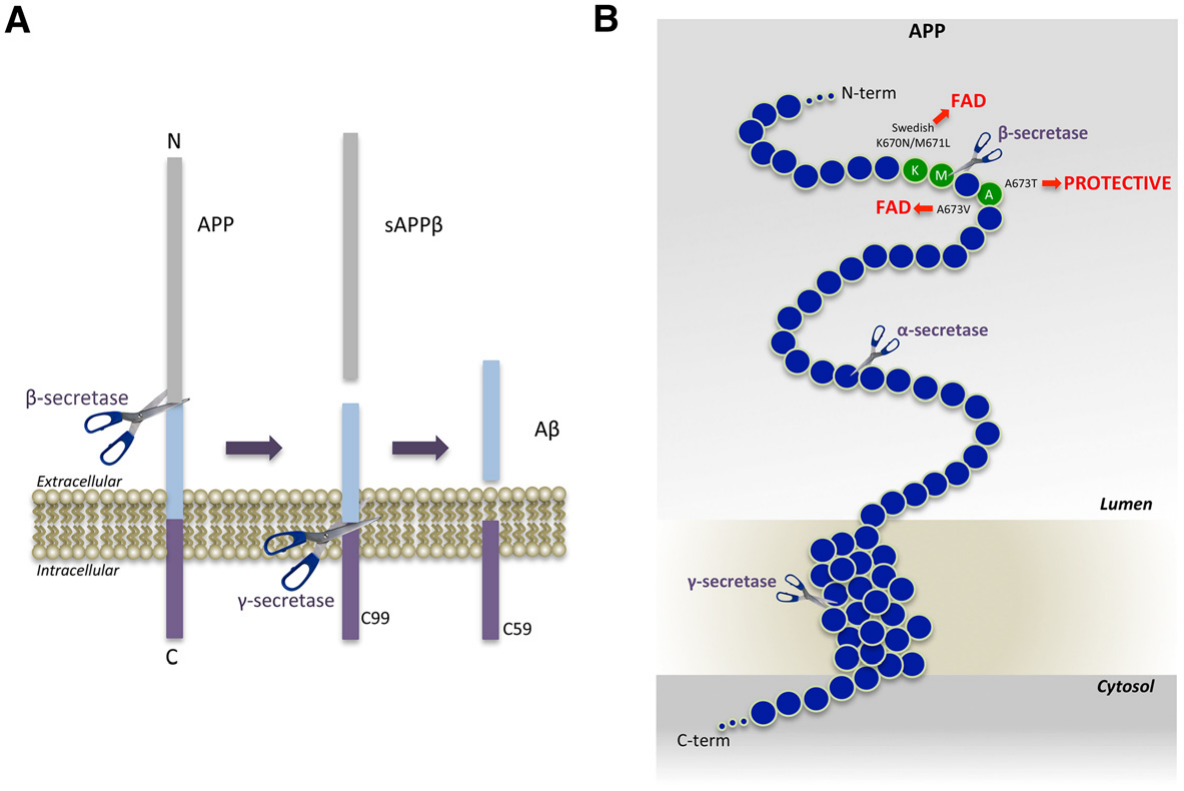
**APP processing and Aβ generation and mutations that affect β-secretase cleavage. A.** APP is a Type-I membrane protein that is sequentially cleaved by two aspartic proteases to generate Aβ. First, the β-secretase enzyme cuts APP (1) to create the N-terminus of Aβ. Two APP fragments are produced: membrane-bound C99 and secreted sAPPβ ectodomain. Second, C99 is cleaved by the γ-secretase enzyme (2) to generate the C-terminus of Aβ. Aβ is then released into the lumen of the endosome and secreted into the extracellular medium. An intracellular domain, C59, is also produced. **B.** The amino acids in and around the Aβ domain of APP are represented as blue circles. Amino acids that affect β-secretase processing of APP in humans are green circles, within which the wild-type residue is identified by the single-letter amino acid code. The K670N/M671L (Swedish) and A673V mutations cause FAD by increasing β-secretase cleavage and Aβ production, while the A673T mutation protects against AD by doing the opposite. All three mutations occur at or within one amino acid of the β-secretase cleavage site. Scissors indicate cleavage sites of the various secretases.

Neurons are the major producers of Aβ in the brain, although glia, in particular astrocytes, may also contribute to Aβ generation, particularly during physiological stress that causes glial activation as happens in AD. The formation of Aβ is a sequential proteolytic process beginning with the cleavage of amyloid precursor protein (APP) by the β-secretase enzyme, which generates the amino (N) terminus of Aβ and yields the membrane bound C-terminal fragment C99 (Figure 1A) [4]. Next, γ-secretase cuts C99 to release Aβ, which is secreted from the cell [5-7]. Interestingly, the γ-secretase cut is imprecise and creates Aβ isoforms of different lengths at the carboxy (C) terminus, of which the longer isoforms are highly associated with AD. Processing of APP by both β- and γ-secretases is necessary for the generation of Aβ, suggesting that inhibition or modulation of either or both of these proteases in the brain should decrease Aβ levels and be beneficial for AD.

Human genetics studies have greatly informed us about AD pathogenesis and strongly suggest that cerebral Aβ accumulation has an essential role in the etiology of AD [2]. Thus far, over 200 autosomal dominant mis-sense mutations have been identified in the genes for APP and presenilin (the γ-secretase catalytic subunit) that are associated with familial AD (FAD). These FAD mutations are highly penetrant and without exception increase either the generation of all Aβ isoforms (total Aβ) or the relative proportion of the 42-amino acid isoform (Aβ42) that is more neurotoxic. Notably, FAD mutations in APP are found very near to the β- and γ-secretase cleavage sites, and these mutations serve to increase APP processing and raise levels of total Aβ or Aβ42 specifically. The so-called Swedish mutation (K670N; M671L) [8] and A673V [9] mutations in APP are particularly compelling, because they are positioned precisely at and only 2 amino acids C-terminal to the β-secretase cleavage site, respectively. These mutations make the cleavage of APP by the β-secretase enzyme more efficient, so greater amounts of C99 and total Aβ are generated (Figure 1B). In contrast, an APP mutation, A673T, has been recently identified that confers protection against AD and cognitive decline in the elderly [10]. This mutation, which occurs at the same position as the A673V mutation that causes FAD, is less efficiently cleaved by β-secretase so that Aβ generation is decreased by ~40% [10-12]. Interestingly, most carriers have one copy of the A673T mutation and likely have a reduction in Aβ production of only ~20%, yet they are still protected against AD. This implies proof-of-principle of the strategy that modest reduction of brain Aβ levels may prevent AD, if started early enough. Additionally, the Swedish, A673V, and A673T mutations together strongly suggest that inhibition of β-secretase cleavage of APP should be beneficial for AD.

## Review

### The identification of β-secretase as β-site APP cleaving enzyme (BACE)

Following the discoveries of Aβ and the first APP mutations that cause FAD, it soon became clear that the β- and γ-secretase enzymes were prime therapeutic targets for the development of small molecule inhibitor drugs for the treatment of AD. Thus, their molecular identities were vigorously pursued. The properties of Aβ generation and secretase activities in cells and tissues led to the development of cell-free and cell-based assays that could be exploited for the identification of the secretases. Subsequently, five groups independently reported the molecular cloning of the β-secretase enzyme, which they variously named β-site APP cleaving enzyme (BACE), Asp2, and memapsin 2 [13-17] (“BACE” has emerged as the most common moniker in the literature). Importantly, all of the groups agreed on the same polypeptide sequence even though they used different experimental approaches to identify the β-secretase, lending strong support for the conclusion that the authentic β-secretase had been cloned.

**Figure 2:**
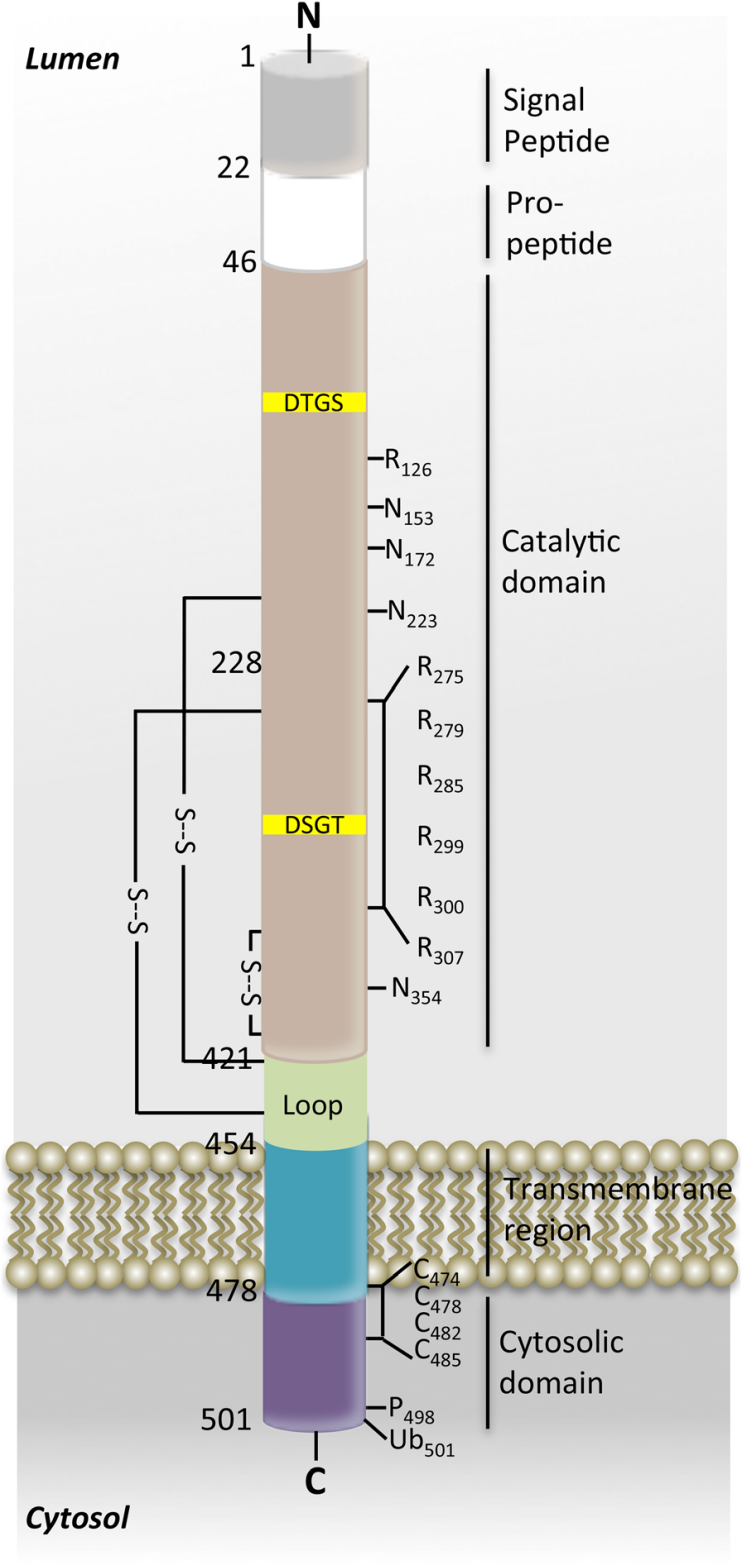
**Primary structure of BACE1.** BACE1 is a 501 amino acid Type-I transmembrane aspartic protease. The various subdomains of BACE1 are indicated by the lines to the right of the structure. Numbers refer to amino acid positions. The two signature aspartic protease active site motifs at positions 93 and 289 are shaded yellow. S--S denote positions of disulfide bridges within the catalytic domain; Ns represents positions of N-linked glycosylation sites; Rs indicates positions of acetylated arginine residues; Cs mark positions of S-palmitoylated cysteine residues; P indicates phosphorylation of serine 498; Ub denotes ubiquitination of lysine 501.

BACE has all the molecular and cellular characteristics that had been previously predicted for the β-secretase in vitro and in vivo [4]. It is a Type I transmembrane aspartic protease of 501 amino acids in length that is closely related to the pepsin family of aspartic proteases (Figure 2). The catalytic domain of BACE harbors two aspartic protease signature motifs of the sequence DTGS and DSGT that come together to form the active site of the enzyme. As required for β-secretase, the BACE active site is topologically oriented on the same side of the membrane as the β-secretase cleavage site in APP. Additionally, the activity of BACE has an acidic pH optimum and the catalytic domain resides within the lumen of acidic intracellular compartments, including endosomes and trans-golgi network (TGN). Moreover, BACE levels are highest in neurons of the CNS, BACE has the correct sequence specificity, and BACE overexpressed in cells cleaves APP and increases Aβ production.

Soon after the discovery of BACE, a homologue, BACE2, was identified that has ~64% amino acid similarity to BACE (henceforth referred to as BACE1) [18]. The extensive degree of homology between the two enzymes suggested that BACE2 might also function as a β-secretase. However, this possibility seemed unlikely because BACE2 is not expressed to a high level in neurons, in contrast to BACE1 [19,20]. Moreover, BACE2 predominantly cleaves APP within the Aβ domain, so that the generation of Aβ is precluded [21-25]. These data, together with the finding that BACE1 null mice are devoid of Aβ (see below), suggest BACE2 is not likely to be a β-secretase in the CNS.

### Physiological functions of BACE1

#### BACE1−/− mice

To justify BACE1 inhibitor drug development efforts, it was necessary to provide in vivo validation that BACE1 is the primary β-secretase enzyme in the brain. To do so, gene targeting in embryonic stem cells was used to produce BACE1 knockout (−/−) mice [26-29]. Initial reports showed that BACE1−/− mice were viable and fertile and did not have detectable abnormalities. Their normal morphology and behavior, tissue histology, and blood cell and clinical chemistry characteristics suggested that BACE1 inhibition as a therapeutic approach for AD might lack mechanism-based toxicities. Additionally, APP overexpressing transgenic mice that also lack the BACE1 gene are devoid of cerebral Aβ, amyloid deposition, and Aβ-associated memory impairments [20,30-33]. Importantly, these data validate BACE1 as the major β-secretase in the CNS and indicate that BACE2 does not compensate for BACE1 loss of function, at least for the production of Aβ. Furthermore, they strongly suggested that BACE1 inhibition should be a safe and effective therapeutic strategy for AD.

Although initial studies of BACE1−/− mice indicated that BACE1 was not required for viability in vivo, further investigations were necessary to elucidate the physiological functions of BACE1 and fully understand the potential for mechanism-based toxicities of therapeutic BACE1 inhibition. For example, BACE1 protein is highly concentrated in presynaptic terminals of CNS neurons [34,35], suggesting that BACE1 has a role in synaptic function. Moreover, in agreement with high BACE1 expression and presynaptic localization in neurons, deeper analyses of BACE1−/− mice have uncovered numerous subtle neuronal phenotypes, such as axon targeting errors [36-38], reduced myelination [39-41], memory impairments [20,30,32,42,43], reduced muscle spindles [44], neurochemical abnormalities [45], alterations in neurogenesis and astrogenesis [46], increased age-related neurodegeneration [47], reduced spine density [48], retinal pathology [49], endophenotypes of schizophrenia [48], and seizures [42,47,50] (Table 1). Future investigations may reveal even more BACE1 null phenotypes. Any of these BACE1 null phenotypes in theory could represent mechanism-based side effects of BACE1 inhibitor drugs in humans, thus raising a note of caution that therapeutic inhibition of BACE1 might not be completely free of toxicity.

**Table 1:** **BACE1 knockout mouse phenotypes**

**Phenotype**	**Putative Substrate**	**References**
Astrogenesis increase; neurogenesis decrease	Jag1	[46]
Axon guidance defects	CHL1	[36-38]
Hyperactivity	NRG1	[29,48]
Hypomyelination	NRG1	[39-41]
Memory deficits	Unknown	[17,30,32,42,43]
Insulin sensitivity enhanced	Unknown	[29,51,52]
Muscle spindle reduction	NRG1	[44]
Neurochemical deficits	Unknown	[45]
Neurodegeneration with age	Unknown	[47]
Postnatal lethality, growth retardation	Unknown	[29]
Retinal abnormalities	VEGFR1	[49]
Schizophrenia endophenotypes	NRG1	[48]
Seizures	Na_v_β_2_	[42,47,50,53]
Spine density reduction	NRG1	[48]
**BACE2 knockout mouse phenotypes**		
**Phenotype**	**Putative Substrate**	**References**
Normal	---	[29]
Pancreatic β cell increase	Tmem27	[54]
Pigmentation abnormalities	PMEL	[55]
**BACE1/2 double knockout mouse phenotypes**		
**Phenotype**		**References**
Similar to BACE1 knockout, except postnatal lethality is enhanced		[29]

#### Substrates of BACE1

**Figure 3:**
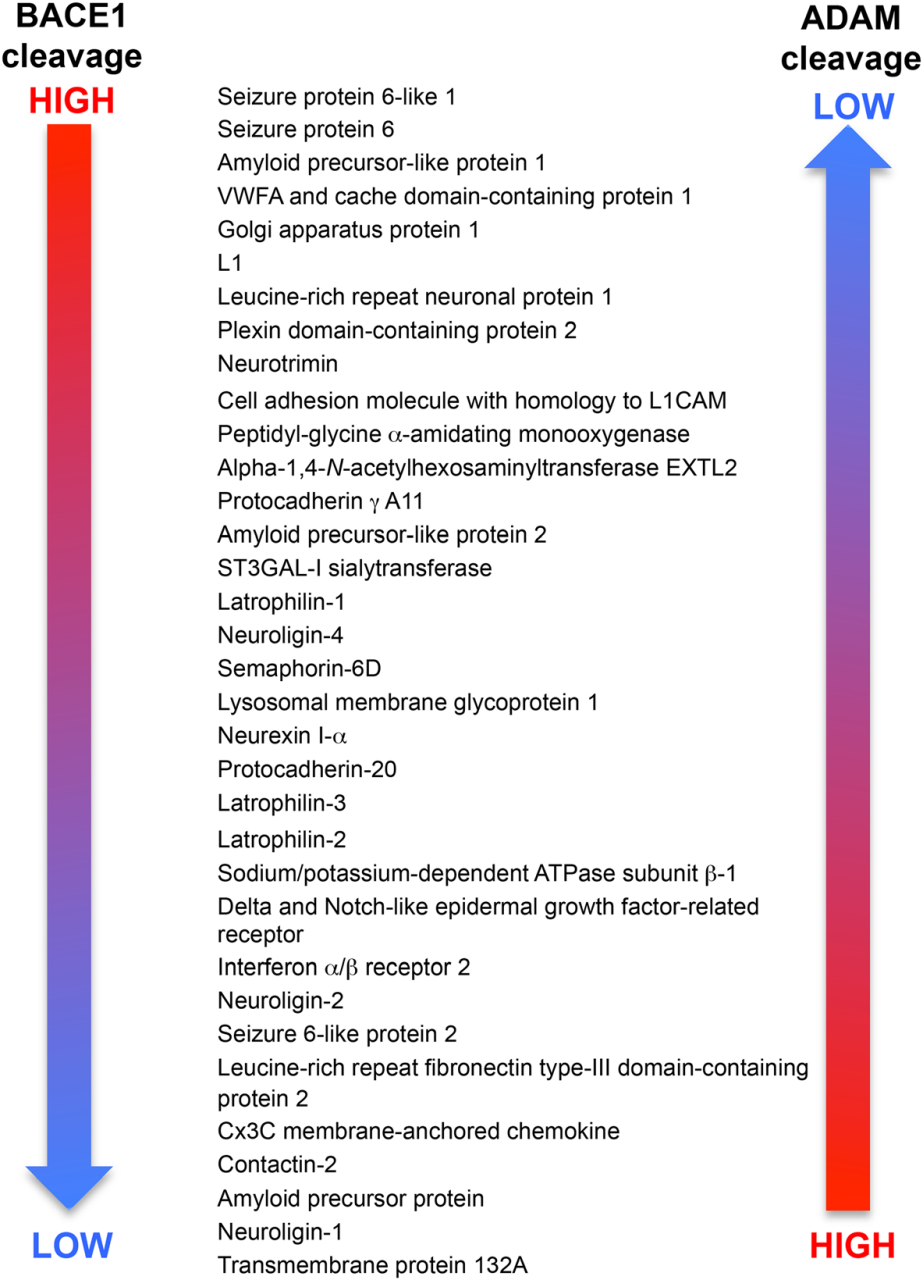
**Neuronal substrates of BACE1.** BACE1 substrates identified in primary cultured neurons are listed from those that are predominantly cleaved by BACE1 (BACE1 cleavage HIGH; top) to those that are processed by BACE1 at a low level (LOW; bottom). These substrates also are cleaved by other proteases in the ADAM family, but the ADAM cleavage preference is opposite to that of BACE1. (Adapted from Table I, Ref. [56]).

The varied phenotypes of the BACE1−/− mice are likely the result of abrogated β-secretase processing of different substrates of BACE1 in addition to APP. Recent proteomic analyses in cultured primary neurons have identified numerous putative BACE1 substrates that have roles in neuronal functions [56,57] (Figure 3). The majority of substrates of BACE1 are, like APP, Type I membrane proteins, while a few, like neuregulin 1 (NRG1), have more complex membrane topologies. Cleavage of most substrates by BACE1 releases an ectodomain fragment that diffuses from the cell in the extracellular milieu. There, it may bind to another molecule on the same (autocrine) or a different (paracrine) cell to affect signal transduction or cell-cell interactions. Perhaps the best studied example is that of BACE1 processing of Type III NRG1, which releases an epidermal growth factor (EGF)-like domain that binds to the ErbB receptor on the Schwann cell for the simulation of myelination [39,40,58,59]. Because of the lack of β-secretase processing, BACE1−/− mice have decreased shedding of the NRG1 EGF domain, which reduces instructive signals to myelinating cells and leads to hypomyelination.

Another example of β-secretase processing of a neuronal substrate involves the cleavage of the neural cell adhesion molecule close homolog of L1 (CHL1) by BACE1. Like APP, CHL1 is a Type I membrane protein, and it has a well-known function in axonal outgrowth and neuronal survival [60,61]. Cleavage of CHL1 by BACE1 liberates a soluble ectodomain fragment that may bind to neuropilin-1 and semaphorin 3A, two molecules that are involved in axonal guidance. Thus, the lack of β-secretase processing of CHL1 might account for the presence of mis-targeted axons that have been reported in the olfactory bulb and hippocampus of BACE1 null mice [38,56,57].

Although decreased β-secretase processing of many BACE1 substrates impairs their function, abrogated cleavage of other substrates may potentiate their role in a physiological process. For example, Jagged 1 (Jag1) is a Type I membrane protein that is a ligand for the Notch receptor, which regulates the differentiation of many cell types in the body. Interestingly, Jag1 is also a BACE1 substrate, and reduced BACE1 cleavage of Jag1 in BACE1−/− mice increases the levels of Jag1 on the cell surface, which causes greater than normal stimulation of Notch activity in the neighboring cell. Consequently, during early development Jag1-Notch signaling is increased [62] in radial glial neural stem cells, which promotes astrogenesis over neurogenesis [46]. As additional BACE1 substrates and functions are discovered, the underlying molecular mechanisms of BACE1 null phenotypes and their implications for mechanism-based toxicities of therapeutic BACE1 inhibition will come into clearer focus.

In addition to cleavage by BACE1, a number of BACE1 substrates undergo ectodomain shedding by proteases in the A Disintegrin and Metalloproteinase Domain (ADAM) family. The extent to which a given substrate is processed by BACE1 verses an ADAM family member varies depending on the substrate (Figure 3). Some substrates are almost exclusively cut by BACE1 (e.g., SEZ6, APLP1), while other substrates are primarily cleaved by the ADAMs (e.g., APP, neuroligin-1) [56,57]. One would predict from these results that potential mechanism-based side effects that arise from therapeutic BACE1 inhibition might derive from deficient processing of substrates that predominantly undergo ectodomain shedding by BACE1 rather than the ADAMs. Conversely, potential toxicities of BACE1 inhibition may be less associated with substrates that are primarily cleaved by ADAM proteases over BACE1.

#### BACE2−/− mice

The significant amino acid similarity shared by BACE1 and BACE2 suggests that it may be challenging to develop BACE1 inhibitors that do not cross-inhibit BACE2. Therefore, the possibility exists that BACE1 inhibitor drugs might also cause BACE2 mechanism-based side effects in addition to those of BACE1. To investigate this question, BACE2−/− mice were produced by gene-targeting. Like BACE1 null mice, the BACE2−/− mice were initially shown to be viable and fertile with no reported phenotype [29]. Moreover, other than enhanced early postnatal lethality, BACE1−/−; BACE2−/− double knockout mice did not have a more severe phenotype than the BACE1−/− single knockouts [29]. These data suggest that cross-inhibition of BACE2 with BACE1 inhibitors might not be associated with enhanced toxicity in the adult after postnatal development is completed.

Although BACE2−/− mice initially were reported to be normal, further investigations have revealed BACE2 loss-of-function phenotypes. Pancreatic β-cells express significant levels of BACE2. Interestingly, BACE2−/− mice have increased β-cell mass and insulin levels, and the mice exhibit enhanced glucose regulation [54]. These phenotypes appear to be the result of abrogated BACE2 cleavage of pro-proliferative Type I transmembrane protein Tmem27, a protein involved in the regulation of β-cell mass. Given these results, inhibition of BACE2 may be beneficial for the treatment of Type 2 diabetes, although further research into this hypothesis is necessary.

In addition to the pancreatic phenotype, BACE2−/− mice on a C57BL/6 genetic background exhibit hypopigmentation that results in a silvery coat compared to the dark coat of wild-type C57BL/6 mice. This phenotype is caused by lack of BACE2 processing of the melanocyte protein PMEL that is expressed in pigment cells of the skin and eye. BACE2 cleavage releases a fragment of PMEL into melanosomes that forms a matrix of amyloid fibrils upon which melanin is deposited [55]. Consequently, abrogated processing of PMEL in BACE2−/− mice leads to abnormal melanosome formation and hypopigmentation. These results suggest the possibility that cross-inhibition of BACE2 by BACE1 inhibitors might cause reduced pigmentation in humans.

### Small molecule BACE1 inhibitor drugs and clinical trials for AD

The extensive validation of BACE1 as the primary β-secretase enzyme in the CNS has spurred vigorous efforts to develop small molecule inhibitors of BACE1 in both academia and industry. The first generation of BACE1 inhibitors consisted of non-cleavable peptide-based transition state analogs designed after the amino-acid sequence in APP at which β-secretase cleaves [15,63]. Typically, these large peptidomimetic molecules are very potent BACE1 inhibitors in vitro, mainly because the large open active site of BACE1 has evolved to bind polypeptide substrates with high affinity. Unfortunately, the peptide-based BACE1 inhibitors did not possess favorable in vivo pharmacological properties, such as oral bioavailability, long serum half-life, or blood–brain barrier (BBB) penetration. As a consequence, investigators have turned toward designing true small molecule BACE1 inhibitor drugs. However, the development of non-peptidic BACE1 inhibitors large enough to bind with sufficient affinity to the enzymatic active site, yet small enough to exhibit satisfactory pharmacokinetics and suitable brain penetration has proven to be very challenging. Moreover, BACE1 inhibitors should have sufficient lipophilicity to cross both plasma and endosomal membranes for gaining access to the vesicle lumen where the BACE1 active site is located.

A crucial advance in small molecule BACE1 inhibitor development came with the first X-ray co-crystal structure of BACE1 with a peptidic BACE1 inhibitor [64]. The BACE1 X-ray structure revealed important inhibitor-enzyme interactions that were exploited in rational drug design efforts. Shortly thereafter, new classes of small molecule BACE1 inhibitors were developed that exhibited improved pharmacological characteristics, including small molecular weight, plasma membrane permeability, and better pharmacokinetics [65,66]. However, most second-generation BACE1 inhibitors were substrates of P-glycoprotein, the ATP-dependent drug efflux pump for xenobiotics in the BBB [67], and therefore could not reach high concentrations in the brain.

More recently, potent third-generation small molecule BACE1 inhibitors have been developed that achieve satisfactory brain penetration and robust cerebral Aβ reduction in preclinical animal models. Innovative diverse and complex drug development approaches have been employed to design current BACE1 inhibitors, which are described in detail in recent reviews [65,66]. Several of these orally bioavailable BACE1 inhibitor drugs have entered into human clinical trials (Table 2). Most are in the early clinical phases and scant information on their progress has been published, although preliminary trial results for three BACE1 inhibitor drugs have been reported at recent conferences and are summarized below.

**Table 2:** **Small molecule BACE1 inhibitors in clinical trials**

**Company**	**Drug**	**Phase**
AstraZeneca/Lilly	AZD3293	Phase 2/3
CoMentis	CTS-21166	Phase 1
Eisai/Biogen Idec	E2609	Phase 2
High Point	HPP854	Phase 1
Janssen/Shionogi	- - - - - -	Phase 1
Lilly	LY2886721	Phase 2*
Merck	MK-8931	Phase 2/3
Novartis	- - - - - -	Phase 1
Pfizer	PF-05297909	Phase 1
Roche	RG7129	Phase 1**
Takeda	TAK-070	Phase 1
Vitae/Boehringer Ingelheim	VTP-37948	Phase 1

*Terminated due to abnormal liver biochemistry.

**Removed from pipeline.

#### LY2886721

The pharmaceutical company Eli Lilly was among the first to develop and test orally bioavailable non-peptidic BACE1 inhibitors in humans. The small molecule BACE1 inhibitor LY2811376 showed satisfactory pharmacokinetic and pharmacodynamic characteristics in preclinical animal models that translated to a Phase 1 clinical trial in humans [68]. However, chronic toxicology studies in rat showing non-clinical non-target associated pathology in retina and brain precluded the clinical development of this molecule. Although it was discontinued, LY2811376 demonstrated the feasibility of developing a potent brain-penetrant orally bioavailable small molecule BACE1 inhibitor and represented the first reported translation of reduced CSF biomarkers of BACE1 cleavage from preclinical animal models to humans.

Lilly advanced a next-generation compound, LY2886721, into Phase 1 and 2 clinical trials to determine its safety and tolerability, pharmacokinetics, and pharmacodynamics. Similar to LY2811376, LY2886721 was a potent orally bioavailable small molecule BACE1 inhibitor that robustly decreased levels of Aβ in the brains of preclinical animal models. However, unlike LY2811376, treatment with LY2886721 did not appear to be toxic to the retina or brain. Forty-seven healthy volunteers were given daily oral doses of either LY2886721 or placebo for 14 days in Phase 1 [69]. Either a multiple ascending dose (5, 15, and 35 mg) or a single dose (70 mg) followed by a multiple ascending dose was performed in two Phase 1 study designs (NCT01227252, NCT01534273). Over the course of the 14-day study, LY2886721 was reported to be safe and well tolerated. Plasma half-life of LY2886721 was ~12 hours, compatible with once per day dosing. Dose-dependent decreases of both plasma and CSF Aβ40 levels resulted from LY2886721 administration. Aβ40 levels in the CSF were decreased up to 74% with the highest dose of LY2886721. Levels of Aβ42 and sAPPβ in the CSF were both reduced to a similar extent as CSF Aβ40 by LY2886721. Interestingly, levels of sAPPα, the α-secretase cleavage product, were increased in CSF [70], an observation that is consistent with BACE1 inhibition, since β- and α-secretase compete for processing of APP. A Phase 1 study of LY2886721 in patients with AD was also conducted (NCT01807026).

The positive Phase 1 trials led to a six-month Phase 2 trial of 35 or 70 mg of LY2886721 dosed orally once per day in 130 patients with prodromal AD, also known as amnestic mild cognitive impairment (MCI), or mild AD (NCT01561430) [71]. Recently, Lilly voluntarily terminated the Phase 2 trial because a small number of subjects that were given LY2886721 developed abnormal liver biochemistries. The company reported that the LY2886721-related liver abnormalities did not appear to be associated with the BACE1 mechanism of action, a conclusion supported by a normal liver phenotype of BACE1−/− mice. It is not uncommon that some small molecules in clinical development are discontinued because of abnormal liver function as a non-target related side effect. Thus, the termination of LY2886721 does not necessarily suggest that BACE1 is not a viable drug target.

#### MK-8931

MK-8931, a small molecule BACE1 inhibitor developed by the pharmaceutical company Merck was tested in 88 healthy volunteers (18–45 years old) as a two-part randomized, double-blind, placebo-controlled Phase 1 clinical trial [72]. Single and multiple (daily for 14 days) oral doses of MK-8931 were analyzed for safety, tolerability, pharmacokinetics, and pharmacodynamics. In healthy volunteers, MK-8931 was well tolerated and no serious adverse events were reported. Determining whether MK-8931 was able to enter the brain and engage its target, the β-secretase enzyme, were primary goals of the study. To do so, biomarkers of BACE1 activity were measured in the CSF, including Aβ40, Aβ42, and sAPPβ, the latter being the BACE1-cleaved ectodomain of APP. MK-8931 markedly reduced levels of Aβ in the CSF in a sustained and dose-dependent manner. A single oral dose of 100 or 550 mg of MK-8931 decreased CSF Aβ40 levels by 75% or 92%, respectively, at 36 hours after dosing. Levels of Aβ42 and sAPPβ in the CSF were also reduced to similar extents. Multiple oral dosing of MK-8931 lowered Aβ levels in the CSF by over 90%. MK-8931 has a plasma half-life of ~20 hours, suggesting that a single daily oral dose may maintain stable drug levels *in vivo*.

A randomized, double-blind, placebo-controlled Phase 1b trial of MK-8931 in 32 mild to moderate AD patients (mean age and Mini-Mental State Examination (MMSE), 73 yrs. and 22, respectively) was also conducted for safety, tolerability, pharmacokinetics, and pharmacodynamics (NCT01496170) [73]. One of three doses (12, 40, or 60 mg) of MK-8931 or placebo was given once each day orally for 7 days and levels of Aβ40, Aβ42, and sAPPβ in the CSF were measured. Similar to the healthy volunteers, MK-8931 strongly decreased levels of Aβ in the CSF in a sustained and dose-dependent fashion. Daily dosing of 12, 40, or 60 mg reduced CSF Aβ40 by 57, 79, or 84%, respectively, and resulted in similar reductions for CSF Aβ42 and sAPPβ. MK-8931 did not appear to cause serious adverse events in the AD patients during the course of the study. Importantly, the MK-8931 Phase 1b results suggest that the pharmacokinetic and pharmacodynamic properties of BACE1 inhibitor drugs are not significantly altered by the presence of high amyloid loads in the brains of AD patients.

Encouraged by the positive results of the MK-8931 Phase 1 and 1b studies, a Phase 2/3 combined clinical trial (the EPOCH study, NCT01739348) was started in late 2012. EPOCH is a 78-week, randomized, placebo-controlled, parallel-group, double-blind clinical trial to evaluate the safety and efficacy of 12 or 40 mg/day oral dosing of MK-8931 versus placebo in mild to moderate AD patients. In Phase 2 the trial will evaluate 200 AD patients and will enroll up to 1,700 patients for Phase 3. Primary efficacy outcomes are the changes from baseline in the Alzheimer’s Disease Assessment Scale Cognitive Subscale (ADAS-Cog) and the Alzheimer’s Disease Cooperative Study-Activities of Daily Living (ADCS-ADL) scores.

A recent interim safety analysis in 200 AD patients treated with MK-8931 for at least 3 months suggested that the drug was well tolerated and that the EPOCH study proceed without changes to the protocol. Enrolment in the trial has continued with up to 1960 patients expected for Phase 3. An additional clinical trial (the APECS study, NCT01953601) has also commenced, consisting of a 104 week randomized, placebo-controlled, parallel-group, double-blind Phase 3 study to evaluate the safety and efficacy of 12 mg or 40 mg per day oral dosing of MK-8931 versus placebo in 1500 patients with MCI. The primary efficacy outcome in APECS is the change from baseline in the Clinical Dementia Rating Scale-Sum of Boxes (CDR-SB) score. Secondary outcome substudies are included in both EPOCH and APECS to measure AD biomarkers, consisting of cortical amyloid load, CSF Aβ and tau, and hippocampal volume. The Phase 3 efficacy studies for EPOCH and APECS are expected to conclude in 2017 and 2018, respectively.

#### AZD3293

The AstraZenica BACE1 inhibitor, AZD3293, was recently tested for safety, tolerability, pharmacokinetics, and effects on plasma and CSF Aβ levels in healthy young (18–55 yr) and elderly (55–80 yr) subjects [74]. Phase 1 randomized, double-blind, placebo-controlled SAD and MAD studies were conducted. In the SAD study (NCT01739647), 1 to 750 mg doses of AZD3293 were administered to 7 young cohorts, while an elderly cohort received 15 mg (8 subjects/cohort). In the MAD study (NCT01795339), multiple once-daily doses of AZD3293 ranging from 15 to 70 mg were administered to 5 cohorts (two elderly) for 2 weeks. AZD3293 was well tolerated with no serious adverse events reported up to the highest dose given (750 mg) in the SAD study. The half-life of AZD3293 was 11–20 hours and thus compatible with once daily dosing. Pharmacokinetic parameters of AZD3293 between elderly and young subjects were indistinguishable. In the MAD study, the 15 or 50 mg doses reduced CSF Aβ40 and Aβ42 concentrations by a constant 50 or 75%, respectively. Additionally, AZD3293 administration produced dose-dependent decreases and increases of sAPPβ and sAPPα concentrations in the CSF, respectively, that had similar timelines as the reductions in CSF Aβ40 and Aβ42 [75]. The Phase 1 studies of AZD3293 in health subjects (NCT01739647) and AD patients (NCT01795339) have been completed, and combined Phase 2/3 trials in 1,551 MCI and mild AD patients are planned (20 mg or 50 mg doses, 104 week duration, AMARANTH trial (NCT02245737)). Recently, AstraZenica and Lilly entered into a partnership to jointly develop AZD3293 for AD.

#### E2609

The drug company Eisai has developed an orally bioavailable small molecule BACE1 inhibitor, E2609, that has shown robust lowering of cerebral Aβ in preclinical and clinical studies. E2609 was first clinically studied in healthy volunteers in randomized, double-blind, placebo-controlled Phase 1 trials [76-78]. A single oral ascending dose (SAD) study (73 subjects) and a 14-day multiple oral ascending dose (MAD) study (50 subjects) tested E2609 in two separate Phase 1 clinical trials (NCT01294540 and NCT01511783, respectively). The SAD study analyzed plasma Aβ levels following E2609 administration ranging from 5 to 800 mg (9 cohorts), while the MAD study measured both plasma and CSF Aβ levels in response to E2609 doses ranging from 25 to 400 mg (5 cohorts). The E2609 plasma half-life of 12–16 hours is compatible with once per day dosing. Each of the two Phase 1 studies showed robust dose-dependent decreases of levels of Aβ in the CSF and/or plasma. CSF Aβ levels were reduced up to 85% at the highest dose of E2609 (400 mg) in the MAD study. Similar decreases in levels of sAPPβ in the CSF were observed, while CSF levels of sAPPα were increased. E2609 appeared to be safe and well tolerated, as no serious adverse events were reported in either Phase 1 study. Eisai has recently completed a Phase 1 trial of E2609 in subjects with MCI or mild AD (NCT01600859), and a Phase 2 clinical trial of E2609 is planned. Recently, Eisai and Biogen Idec entered into a partnership to jointly develop E2609 for AD.

#### Alternative therapeutic approaches for BACE1 inhibition

Although small molecules that directly inhibit BACE1 enzyme activity are leading therapeutic approaches, potential alternative strategies to reduce BACE1 processing of APP are being explored. As noted, BACE1 levels are significantly elevated in AD brain and might accelerate the production of Aβ. Therefore, approaches to lower and normalize BACE1 levels in the brain might slow AD progression and avoid possible untoward side effects caused by direct BACE1 enzyme inhibition. Consequently, efforts are underway to elucidate the mechanisms of BACE1 elevation in AD in order to identify drug targets that could block the BACE1 increase when inhibited. BACE1 undergoes complex regulation at the transcriptional, translational, and post-translational levels, all of which appear to have a role in elevating BACE1 levels and activity in AD [79-81]. Much evidence suggests that BACE1 is a stress response protease that is increased by oxidative stress, inflammation, hypoxia, and trauma, among other insults that occur in AD [79,82,83]. Even Aβ itself increases BACE1 levels in neurons [84,85], suggesting a vicious pathogenic cycle whereby Aβ could accelerate its own production through BACE1 elevation. Which, if any, of these complex multi-layered regulatory mechanisms might yield therapeutic strategies for lowering BACE1 levels in AD is unclear, but continuing research in this important area may reveal promising new AD drug targets in the future.

Another class of alternative therapeutic strategy for BACE1 inhibition involves immunotherapy approaches to reduce BACE1 processing of APP. The first of these strategies employs antibodies directed against the β-secretase cleavage site of APP that sterically block access of the BACE1 active site to APP [86,87]. These anti-β-site APP antibodies decrease Aβ production cultures cells and when injected i.v. reduce amyloid plaque pathology in the brains of APP transgenic mice [88]. Other immunotherapy approaches include anti-BACE1 antibodies that are not directed against the active site but instead target an exosite on the surface of the BACE1 catalytic domain that can allosterically regulate enzyme activity [89,90]. This exosite is located on structurally adjacent regions of the C, D, and F loops of the enzyme [91]. Exosite antibody binding to BACE1 alters structural features and dynamic characteristics near the substrate binging cleft of the enzyme. Additionally, transport of BACE1 antibodies across the BBB has been facilitated by engineering one arm of the antibody to recognize transferrin receptor (TfR), which shuttles transferrin across the BBB for the delivery of iron into the brain [92,93]. These bispecific BACE1-TfR antibodies accumulate in the brain and reduce endogenous Aβ levels in mice to a much greater extent than monospecific BACE1 antibodies. Moreover, TfR bispecific antibodies could be useful for treating other neurologic diseases amenable to immunotherapy. These antibody approaches are currently in preclinical phases.

### Unanswered questions that are relevant to BACE1 inhibitor clinical trials

Fifteen years after the discovery of the β-secretase enzyme, the challenges of developing brain-penetrant BACE1 inhibitors have been accomplished and human clinical trials are underway. This promising development raises hopes that disease-modifying therapies employing BACE1 inhibition for AD are within reach. However, important questions concerning therapeutic goals and outcomes of these trials remain to be answered:

#### What degree of BACE1 inhibition will be needed to achieve efficacy?

The level of BACE1 inhibition required for efficacy in turn should depend on how much Aβ lowering is necessary and at what stage of AD to treat (questions discussed further below). The recently discovered A673T APP mutation that protects against AD [10] suggests that reducing cerebral Aβ production by only a modest amount (~20%) could be preventative, if started before significant amyloid accumulation. As discussed above, the leading BACE1 inhibitors currently in clinical trial are capable of this relatively small Aβ decrease. How BACE1 inhibition translates to Aβ reduction in the brain is difficult to estimate, although some insight into this question may be gained by considering experiments in BACE1 knockout mice. Heterozygous BACE1+/− mice that model 50% therapeutic inhibition of BACE1 exhibit ~20% lowering of cerebral Aβ levels in APP transgenic mice [20,33]. Importantly, BACE1+/− mice appear to be normal, so 50% BACE1 inhibition may circumvent mechanism-based side effects yet provide sufficient Aβ reduction for efficacy.

As suggested by the protective A673T mutation, a therapeutic approach that reduced BACE1 activity and Aβ levels by ~50% and ~20%, respectively, would probably need to start before major amyloid deposition and be maintained for the remainder of life to prevent or delay the onset of AD. However, inhibiting BACE1 by more than 50% could be required if significant amyloid plaque load is present in the brain at the beginning of treatment. Still, the possibility exists that no level of BACE1 inhibition, no mater how strong, would be able to slow the progression of AD once a certain threshold of amyloid burden is reached. At present, these arguments are all speculative, as the levels of BACE1 inhibition and Aβ reduction necessary for efficacy in humans are as yet unknown, although insight into these parameters might be gleaned following analysis of the results from the ongoing clinical trials.

It is important to note that cerebral BACE1 levels in AD patients are increased by several fold over those in normal individuals [94-97]. Both BACE1 and APP accumulate in swollen dystrophic neurites that surround amyloid plaques [34,98,99], suggesting increased peri-plaque Aβ production that might accelerate amyloid deposition and induce a vicious pathogenic cycle [100]. If so, normalization of BACE1 activity in peri-plaque dystrophic neurites may represent a modest but potentially efficacious therapeutic goal of BACE1 inhibition. However, elevated concentrations of BACE1 around plaques might necessitate the administration of very high BACE1 inhibitor doses in order to significantly reduce peri-plaque Aβ generation, if the amyloid burden is great.

#### What stage of AD should we administer BACE1 inhibitors?

Cerebral Aβ accumulation has a crucial early role in AD pathogenesis, as suggested by over 200 FAD mutations [2]. Amyloid deposition appears to begin more than a decade before the manifestation of cognitive deficits and the clinical diagnosis of AD [101-103]. Aβ-lowering BACE1 inhibitors are likely to be most effective as a prevention strategy when administered early in the course of AD, before significant cerebral amyloid accumulation and neurodegeneration. Thus, BACE1 inhibitors are analogous to the cholesterol-lowering statin drugs for the prevention of heart disease: once significant amounts of cholesterol have deposited in coronary arteries and major injury to the heart has occurred, statin administration is unable to reverse the damage and provide much benefit for the patient. AD prevention trials will necessarily involve the enrollment of thousands of subjects, last for years, and incur enormous costs. As a result, AD prevention trials might be most feasible in the context of joint government-industry collaborations, such as those being conducted or planned by the Anti-Amyloid Treatment in Asymptomatic Alzheimer’s Disease (A4) trial, Alzheimer’s Prevention Initiative (API), and Dominantly Inherited Alzheimer Network Trials Unit (DIAN TU). Some AD prevention trials are enrolling cognitively normal individuals that are genetically at high risk for developing AD who carry autosomal dominant FAD mutations (DIAN TU trial) or are homozygous for the apolipoprotein E ε4 allele (API trial). If BACE inhibitors are as well tolerated in chronic dosing for AD as the statins are for lowering serum cholesterol to prevent heart disease, then treating pre-symptomatic at-risk individuals for AD prevention is warranted.

Current BACE1 inhibitor trials have enrolled mild and moderate AD or mild cognitive impairment (MCI), the latter of which progresses to AD at a rate of ~10-15% per year [104]. A major advance has been the development of amyloid positron emission tomography (PET) imaging and CSF Aβ42 measurement as biomarkers for the diagnosis of prodromal AD [105,106]. Individuals that exhibit significant amyloid load by PET or have reduced CSF Aβ42 concentrations are likely to develop AD, even though they appear cognitively normal at the time of testing. Due to the unavailability of amyloid PET or CSF Aβ42 biomarker testing at the time, past Aβ immunotherapy trials were unable to exclude subjects that did not have cerebral amyloid accumulation, thus leading to increased variability in cognitive outcome results and ultimately contributing to the frank failure of these clinical trials [107]. In contrast, the BACE1 inhibitor clinical trials are enrolling only subjects that are positive by amyloid PET or CSF Aβ42, which should decrease data variability and increase the probability of observing statistically significant differences in cognition between drug and placebo groups. Periodic amyloid PET or CSF Aβ42 testing will be conducted to monitor target engagement and amyloid accumulation over the courses of the trials. Cognitive performance will also be tested, as this measure is the gold standard for efficacy in past AD clinical trials of approved palliative drugs that treat memory symptoms. However, as noted above, amyloid deposition appears to start years before memory deficits are detected with current tests of cognition. Thus, it might be challenging for BACE1 inhibitors to alter the trajectory of AD once a large amount of amyloid has accumulated in the brain, at least regarding the reduction of cognitive decline.

It is hoped that the levels of BACE1 inhibition and Aβ reduction necessary for disease modification could be deduced from data collected at the conclusion of the current clinical trials. Pharmacodynamic models developed from this future data might assist in the estimation of the level of BACE1 inhibition required to achieve efficacious Aβ reduction for a given cerebral amyloid load and level of cognitive impairment. These models could also be useful for the design of future primary and secondary AD prevention trials in pre-symptomatic individuals. At this time, the relationships between BACE1 inhibition, Aβ reduction, amyloid load, and cognitive status are not sufficiently well understood to develop accurate pharmacodynamic models for estimating the levels of BACE1 inhibition needed at a given stage of asymptomatic or symptomatic AD.

#### Will treatment with BACE1 inhibitors cause mechanism-based side effects?

Although BACE1−/− mice were initially reported to be normal, recent studies have identified over a dozen BACE1 null phenotypes and substantially more BACE1 substrates (Table 1, Figure 3), suggesting therapeutic BACE1 inhibition might cause mechanism-based toxicities. That said, it is unclear to what extent BACE1 null phenotypes in mice are able to model potential BACE1 inhibitor side effects in humans, for several reasons. First, BACE1 null phenotypes could relate to functions of BACE1 either during development or in adulthood, since BACE1−/− mice lack BACE1 from conception. For example, the major proportion of myelination occurs during development and is completed when adulthood is reached [108], indicating that hypomyelination as a result of abrogated BACE1 processing of NRG1 in BACE1−/− mice is a developmental phenotype. Consequently, BACE1 inhibition in the adult might not have an impact on myelination, unless re-myelination following injury becomes necessary. In contrast, neurogenesis and axon guidance are ongoing processes that occur in specific neuronal subpopulations that regenerate throughout life [38,46], suggesting the possibility that BACE1−/− abnormalities in neurogenesis and axon targeting are adult phenotypes and that BACE1 inhibitor treatment might lead to similar defects. Additionally, it is possible that compensation from other proteases during development could mitigate the effects of BACE1 null mutation, in which case BACE1 inhibitor treatment in humans might have more severe side effects than indicated by BACE1−/− mice. Given these arguments, comprehensive analyses of BACE1−/− mice should help to parse developmental verses adult BACE1 null phenotypes for the estimation of BACE1 inhibitor side effect risk.

The risk of BACE1 mechanism-based toxicities will depend in large part on the degree of therapeutic BACE1 inhibition. At one extreme, BACE1−/− mice model 100% BACE1 inhibition, but this level of inhibition will never be achieved by BACE1 inhibitor treatment in practice, thus reducing the chance of side effects. However, AD patients are elderly and often frail, thus increasing the risk of serious adverse events caused by BACE1 inhibition. Moreover, BACE1 inhibitors must be chronically administered, necessitating a high level of safety. Ongoing and future BACE1 inhibitor clinical trials will ultimately answer these questions. It is anticipated that a therapeutic window will be discovered in which an empirically determined range of BACE1 inhibitor doses can balance tolerable mechanism-based side effects with sufficient reduction of cerebral Aβ levels for efficacy.

The statins are useful as a group for modeling the clinical development of BACE1 inhibitors, as indicated by the fact that the statin clinical trials determined a therapeutic dose window of HMG Co-A reductase inhibitor that effectively decreased serum cholesterol levels to prevent heart disease in the presence of tolerable side effects. We are now in the early phases of this clinical development model for BACE1 inhibitors. Regardless of the final outcomes of the current BACE1 inhibitor trials, invaluable knowledge will be gained about the quantitative and temporal relationships between BACE1 inhibition, Aβ reduction, amyloid burden, and cognitive function in humans, which will be used for future clinical development of BACE1 inhibitors for AD.

## Conclusions

As the β-secretase enzyme that initiates the production of Aβ, BACE1 is a key therapeutic target for AD. The protective A673T APP mutation in humans and genetic deletion of BACE1 in mice both decrease Aβ generation via reduced β-secretase processing of APP, providing strong proof of concept that BACE1 inhibition should be efficacious for AD. However, BACE1 null mice exhibit multiple complex neurological phenotypes (Table 1), suggesting that BACE1 inhibitor drugs might cause mechanism-based side effects involving hypomyelination, seizure, axon guidance defects, memory deficits, neurogenesis abnormalities, and neurodegeneration, and potentially others, resulting from insufficient BACE1 processing of a growing list of BACE1 substrates in neurons. Which, if any, of these BACE1 null phenotypes model BACE1 inhibitor side effects in humans remains to ne determined.

Despite the challenges of BACE1 inhibitor drug development over the past 15 years since the discovery of BACE1, the recent introduction of several BACE1 inhibitors into clinical trials has refocused attention on this promising therapeutic approach for AD. To date, Merck’s MK-8931 has advanced the farthest to Phase 2/3, while the other drugs including those from AstraZeneca, Eisai, and Pfizer, among others, are in Phases 1 and 2. These compounds are potent, achieving up to ~90% CSF Aβ reduction. Additionally, they are well-tolerated for the most part, although two BACE1 inhibitors have recently been terminated due to toxicity that might not be related to the BACE1 mechanism of action.

The most challenging questions for BACE1 inhibitor clinical development concern the level of BACE1 inhibition and the stage of AD at which to treat for optimal efficacy. Hypothetical arguments based on the A673T APP mutation and BACE1+/− mice suggest that ~50% BACE1 inhibition might achieve ~20% Aβ reduction, which could prevent AD if begun well before significant amyloid deposition. However, it is unclear whether any level of BACE1 inhibition can be effective if major amyloid accumulation is present in the brain. Amyloid PET imaging, CSF Aβ42 measurement, and other biomarker studies suggest that amyloid deposition starts years, even decades, before the clinical diagnosis of dementia. Moreover, the relationships between amyloid burden and cognitive impairment are not sufficiently well understood to determine the stage of AD that BACE1 inhibitor treatment would be most effective. Results from ongoing biomarker studies, future treatment and prevention trial, and pharmacodynamic modeling are expected to establish the appropriate level of BACE1 inhibition and stage of AD for optimal efficacy. Like the statins for hypercholesterolemia, the hope is that a therapeutic window of BACE1 inhibitor doses might be found that reduces cerebral Aβ levels enough for efficacy, yet maintains sufficient BACE1 activity for the avoidance of side effects. When eventually published, the results of the current BACE1 inhibitor clinical trials will prove invaluable for solving these important questions. We are at a crucial juncture in BACE1 inhibitor drug development, and the therapeutic potential of BACE1 inhibition for AD will be definitively answered in the not too distant future.

## Abbreviations

AD: Alzheimer’s disease
APP: Amyloid precursor protein
Aβ: β-amyloid peptide
BACE1: β-site APP cleaving enzyme 1
C99: Carboxy-terminal 99 amino acid fragment of APP generated by BACE1 cleavage
sAPPβ: Amino terminal ectodomain fragment of APP generated by BACE1 cleavage
